# Health risk assessment and source apportionment of potentially toxic metal(loid)s in windowsill dust of a rapidly growing urban settlement, Iran

**DOI:** 10.1038/s41598-022-21242-z

**Published:** 2022-11-17

**Authors:** Reyhane Madadi, Sedigheh Mohamadi, Mohammad Rastegari, Abdolreza Karbassi, Md. Refat Jahan Rakib, Mayeen Uddin Khandaker, Mohammad Rashed Iqbal Faruque, Abubakr M. Idris

**Affiliations:** 1 Environmental Research Laboratory, School of Civil Engineering, Iran University of Science and Technology, Tehran, Iran; 2 Faculty of Science and Engineering, Southern Cross University, Lismore, NSW 2480 Australia; 3 School of Environment, College of Engineering, University of Tehran, Tehran, Iran; 4 Department of Fisheries and Marine Science, Faculty of Science, Noakhali Science and Technology University, Noakhali, Bangladesh; 5 Centre for Applied Physics and Radiation Technologies, School of Engineering and Technology, Sunway University, 47500 Bandar Sunway, Selangor Malaysia; 6 Department of General Educational Development, Faculty of Science and Information Technology, Daffodil International University, DIU Rd, Dhaka, 1341 Bangladesh; 7 Space Science Centre (ANGKASA), Institute of Climate Change (IPI), University Kebangsaan Malaysia (UKM), 43600 Bangi, Selangor D. E Malaysia; 8 Department of Chemistry, College of Science, King Khalid University, Abha, 61421 Saudi Arabia; 9 Research Center for Advanced Materials Science (RCAMS), King Khalid University, Abha, 61421 Saudi Arabia

**Keywords:** Biochemistry, Biological techniques, Ecology, Zoology

## Abstract

Rapid industrialization and urbanization have resulted in environmental pollution and unsustainable development of cities. The concentration of 12 potentially toxic metal(loid)s in windowsill dust samples (n = 50) were investigated from different functional areas of Qom city with the highest level of urbanization in Iran. Spatial analyses (ArcGIS 10.3) and multivariate statistics including Principal Component Analysis and Spearman correlation (using STATISTICA-V.12) were adopted to scrutinize the possible sources of pollution. The windowsill dust was very highly enriched with Sb (50 mg/kg) and Pb (1686 mg/kg). Modified degree of contamination (mC_d_) and the pollution load indices (PLI_zone_) indicate that windowsill dust in all functional areas was polluted in the order of industrial > commercial > residential > green space. Arsenic, Cd, Mo, Pb, Sb, Cu, and Zn were sourced from a mixture of traffic and industrial activities, while Mn in the dust mainly stemmed from mining activities. Non-carcinogenic health risk (HI) showed chronic exposure of Pb for children in the industrial zone (HI = 1.73). The estimations suggest the possible carcinogenic risk of As, Pb, and Cr in the dust. The findings of this study reveal poor environmental management of the city. Emergency plans should be developed to minimize the health risks of dust to residents.

## Introduction

The fast growth of population and the overlapping of urban and industrial boundaries have deteriorated urban sustainability in developing countries^1,2^. Environmental pollution inevitably arises from industrialization (like mining and smelting operations) and urbanization (like construction and traffic). Since dust contains contaminants like potentially toxic metal(loids) (PTMs), it has been used as an indicator of urban pollution in the last decade.

Ingestion, inhalation, and dermal contact are considered as the main routes of human exposure to dust pollutants^3^. Short/long-term exposure to contaminated dust can threaten human health at different levels (from allergic reactions to cancer diseases)^4^. For instance, medical studies observed that As inhalation during lung development in children can lead to functional and structural alterations due to decreasing club cell protein 16 (CC16)^5^. Oral exposure to Pb adversely affects the central nervous system and neurobehavioral development^6^.

Various factors, such as the city's functional zones, influence the type and concentration of PTMs in urban dust. As an example, mining and industrial activities adjacent to urban areas significantly affect the contents of PTMs in urban dust. As reported by Long et al.^7^, urban dust around industrial areas (iron smelting and steel plant) contained higher amounts of PTMs (Cr, V, Cd, Pb, and Zn) than the other functional areas inside Panzhihua city, China. Tian et al.^8^ observed that in mining zones a high ratio of road dust originated from mining particles distributed via wind.

Dust pollution may stem from multiple sources and travel miles away; thus, it is a formidable challenge to pinpoint its exact source. Several studies have examined road and street dust for assessing pollution levels and they generally attribute the source of dust pollution to traffic emissions^9,10^. Heidari et al.^11^ described traffic emissions as the main contributor to health risks and source of dust pollution in Bandar Abbas suburb. Dytłow and Górka-Kostrubiec^12^ identified that the level of pollution in dust correlates with moving vehicles in Warsaw, Poland.

A possible way to reduce dust contamination is to stop its dispersion from the source. Instead of street dust, windowsill/window dust might be a better choice for determining the source of pollution. Windowsill dust comes from a variety of sources, such as soil erosion and industrial emissions^13^, and it represents a more acceptable level of risk to residential health^2^ by influencing the quality of indoor air. A study in central China found higher concentrations of PTMs in windowsill dust compared to the surface soil in the villages surrounding an industrial zone^2^. Current researches focus on road dust (like traffic nodes and highways) to assess the influence of PTMs on urban residential health, whilst the understanding of PTMs in the other exterior dust samples (such as windowsills) remains seriously incomplete.

This study aims to scrutinize the contamination level and spatial distribution of PTMs in windowsill dust regarding functional sectors (industrial, residential, commercial, and greenspace). For this purpose, we utilized Qom city, Iran as an example of a city with a high rate of urbanization. Mining activities and related industries (smelting and alloy production) in Qom have invited many immigrants, resulting in unsustainable urban growth^1,14^. This work can be considered as the first attempt to: (1) discuss the contamination level and distribution pattern of PTMs, (2) identify possible sources of pollution, and (3) evaluate the potential non-carcinogenic/carcinogenic risks of PTMs in windowsill dust from four functional areas of Qom city, Iran.

## Materials and methods

### Study area

Qom is located 120 km south of Tehran, Iran (34° 26′ N to 34° 47′ N, 50° 41′ E to 51° 23′ E), with an area of approximately 218.14 km^2^ and a population of about 1.25 million. Qom city had the highest rate of unsustainable urbanization during 2006–2016^1^ and it is among the cities with the highest growth rate (3.1%) in Iran. Qom is located near Iran’s main transportation highway connecting Tehran to industrial provinces. Additionally, it is the second holy city in Iran (after Mashhad city) and has a range of tourist attractions.

Qom city is located near a desert with limited access to water resources^15^. In recent years, the city is threatened with climate changes including higher temperatures, dust storms, soil erosion, groundwater loss, and desertification^16^. The climate of the city is semi-arid with average annual precipitation and temperature of 133 mm and 19 °C, respectively. The dominant wind comes from the west in cold seasons, while in summer and spring, the east wind coming from deserts (located in the vicinity of the city) causes dusty storms toward the city^16^. The area is enriched with natural resources including minerals (such as metals, clay, and lime) and building materials. Geologically, Qom is part of the “Central Basin of Iran’s Plateau^17^.” Soil mineralogy is dominated by lithic fragments, quartz, feldspars, zeolite, magnetite, and calcite^17^. Some features of lithofacies include coliform manganese oxides (occurring alongside iron oxides), prevalent secondary gypsum cement, silicic veins, and space copper mineralization (mostly malachite)^17^.

**Figure 1:**
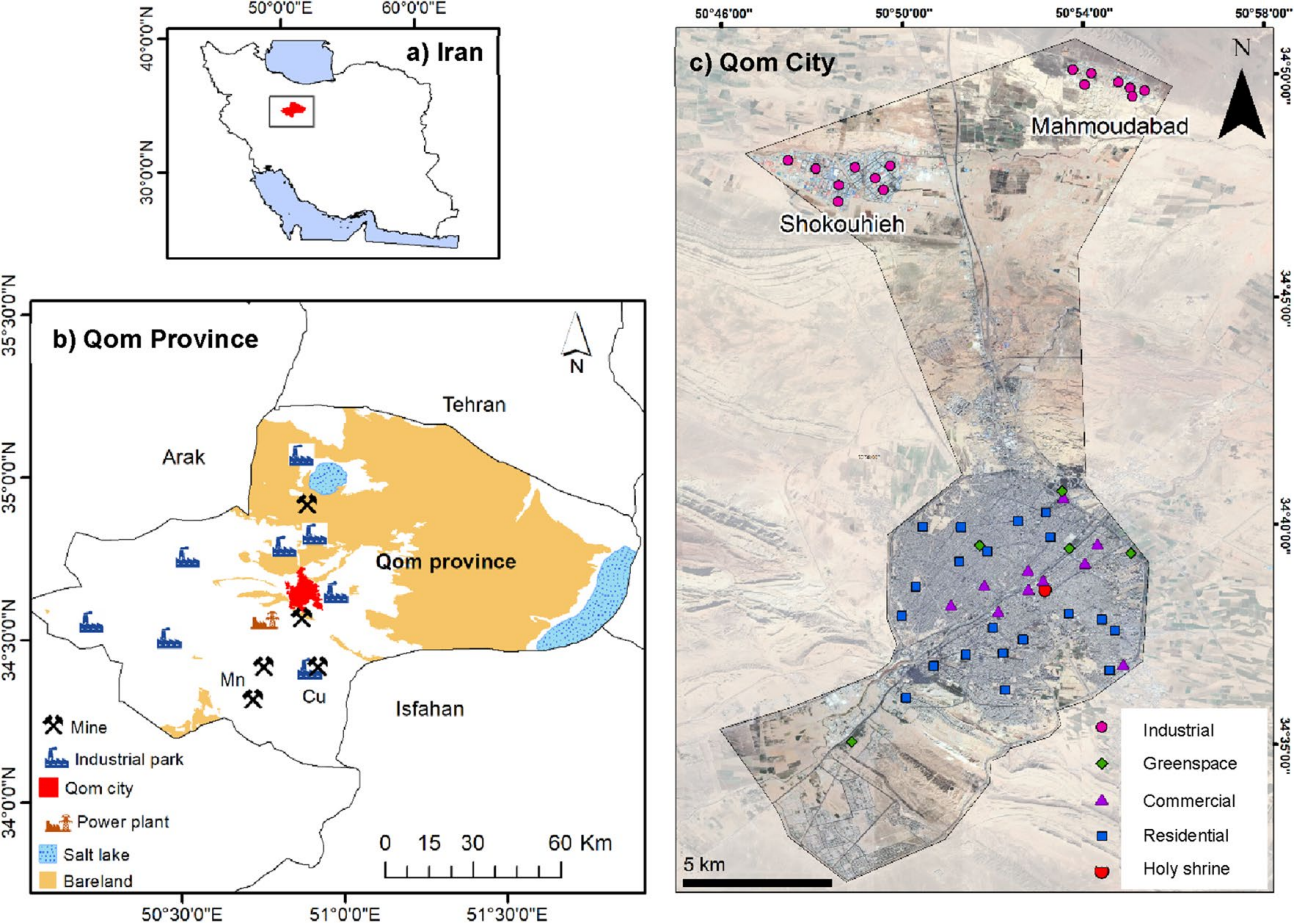
Study area and sampling stations of windowsill dusts, Qom, Iran. This map was constructed using ArcGIS version 10.3. (https://www.esri.com/en-us/arcgis/products/arcgis-desktop/overview).

Two main industrial regions, named Shokouhieh and Mahmoudabad, are located in the northern of Qom (Fig. 1). About 72 active mining companies (out of 102 detected mining areas) exist around the city. Venarj manganese (Mn) mining site—the largest Mn mine in Iran—and Kahak copper (Cu) mining site are located in the southern of Qom city (about 30 km away).

### Sample collection

In this study, a total of 50 windowsill dust samples (Fig. 1) were collected from different functional sectors: residential (20), commercial (10), greenspace (5), and industrial (15). The sampling time took place during two weeks in October 2020, considering a sunny month before and during sampling. In all stations, the geographical coordinates and general information were recorded. At each station, four to six dust samples were collected at a maximum distance of 10 m from windowsills with 1–2 m high from the ground. Approximately 50 g of composite dust samples collected with polyethylene brush were used as the representative sample. Each sample was labeled and stored in a sealed polyethylene bag at a temperature of less than 4 °C.

### Chemical analysis

The dust samples were air-dried at room temperature, then sieved through a 220 mesh (63 μm). This site was selected in this study for elemental analysis as those particles of urban dust are easily re-suspended and can be inhaled through the nose or mouth during breathing. As well, finer particles of dust contain higher toxic elements and are susceptible to adverse environmental and health effects.

To assess the total PTMs content, 1 g of each sample was digested using four acids (5 mL HNO_3_, 5 mL HCl, 15 mL HF, and 2.5 mL HClO_4_) at 220 °C for 4 h. After digestion, the extracts were filtered using filter paper No. 42 and diluted to a volume of 50 mL. The concentration of PTMs (As, Cd, Co, Cr, Cu, Fe, Mn, Mo, Ni, Pb, Sb, and Zn) was measured using inductively coupled plasma atomic emission spectroscopy (ICP-AES, Agilent 735) in Zarazma Mineral Studies Co. (Tehran, Iran). Detection limits are given in Table S1.

### Quality assurance/quality control (QA/QC)

The chemicals and reagents used in this study were of analytical grade (Merck). Blank samples and certified reference material (MESS-3) from the Institute for National Measurement Standards, National Research Council of Canada (NRC) were applied for quality control of PTMs analyses. All samples, blanks, and the certified reference material were analyzed in duplicate. All the measurements were performed in three replicates with a relative standard deviation (RSD) of less than 5%. The recovery percentages of certified reference material ranged from 94 to 106% (Table S2).

### Statistical analysis

Using the Kolmogorov–Smirnov (K–S) test, the data were evaluated for normality. Spearman’s correlation coefficient was utilized to measure the relationship between PTMs in urban dust. Principal component analysis (PCA) was conducted to identify sources of PTMs in urban dust. PCA was carried out using the rotation method of Varimax for an easier interpretation of the components. The statistical analyses were carried out using STATISTICA (V.12) and OriginPro (V.9.8.5) software.

### Pollution assessment

#### Enrichment factor (EF)

The enrichment factor (EF) as a suitable index in assessing PTMs enrichment in urban dust and the possible effects of human activities on the concentration of PTMs is calculated using the following equation (Eq. 1):

1 $$EF = [(C_{n} / C_{ref} )]_{sample} / [(C_{n} / C_{ref} )]_{background}$$

where C_n_ is the concentration of target PTM and C_ref_ is the concentration of iron as a reference element. The background used in this study was the PTMs concentration in the Upper Continental Crust (UCC)^18^. Five categories are defined for EF that are listed in Table S3.

#### Geo-accumulation index (I_geo_)

This index has been widely used to assess PTMs pollution levels in dust, soil and sediment with respect to the geochemical background levels. The I_geo_ can be calculated according to the following equation (Eq. 2):

2 $$I_{geo} = \log_{2} (C_{n} /1.5B_{n} )$$

The I_geo_ index categorizes the pollution level into seven classes, from unpolluted levels to extremely polluted levels (Table S3).

#### Modified degree of contamination index (mC_d_)

The modified degree of contamination (mC_d_), with seven categories (Table S3), was developed by Abrahim and Parker^19^ is calculated as follows (Eqs. (3) and (4)):

3 $$mC_{d} = \left( {\mathop \sum \limits_{i = 1}^{n} C_{f}^{i} } \right)/n$$

4 $$C_{f}^{i} = C_{n}^{i} / C_{b}^{i}$$

where ${\text{C}}_{{\text{f}}}^{{\text{i}}}$ is the contamination factor of PTM_i_, ${\text{C}}_{{\text{n}}}^{{\text{i}}}$ and ${\text{C}}_{{\text{b}}}^{{\text{i}}}$ correspond to the concentration of PTM_i_ in sample and background concentrations, respectively, and n is the number of analyzed elements.

#### Pollution load index (PLI)

The pollution load index (PLI) indicates the overall contamination level of PTMs. The PLI_zone_ is also used for comparing different zones (in this study with four land-use types). The PLI and PLI_zone_ are described in Eq. 5 and Eq. 6:

5 $$PLI = \sqrt[n]{{C_{f}^{1} \times C_{f}^{2} \times \cdots \times C_{f}^{n} }}$$

6 $$PLI_{zone} = \sqrt[m]{{PLI_{1} \times PLI_{2} \times \cdots \times PLI_{m} }}$$

PLI and PLI_zone_ have two classifications: unpolluted (PLI < 1) and polluted (PLI > 1).

### Risk assessment

#### Potential ecological risk index (PERI)

The potential ecological risk index (PERI) to evaluate the environmental risks from PTMs is revealed in Eq. 7:

7 $$RI = \mathop \sum \limits_{i = 1}^{n} E_{r}^{i} = \mathop \sum \limits_{i = 1}^{n} T_{r}^{i} \times C_{f}^{i}$$

where ${\text{E}}_{{\text{r}}}^{{\text{i}}}$ is the ecological risk of each element,${\text{ T}}_{{\text{r}}}^{{\text{i}}}$ is the toxic-response factor of each PTM. ${\text{T}}_{{\text{r}}}^{{\text{i}}}$ values for Cd, As, Pb, Cu, Ni, Cr, and Zn are 30, 10, 5, 5, 5, 2, and 1, respectively. ${\text{C}}_{{\text{f}}}^{{\text{i}}}$ represents the contamination factor of PTM_i_. Different PERI classifications are presented in Supplementary Table S3.

#### Non-carcinogenic risk

The non-carcinogenic health risk of PTMs can be estimated using the hazard index (HI) which is the summation of the hazard quotient (HQ). HQ is defined as the ratio of average daily dosage (ADD, mg/kg.day) to that of reference dose (RfD). In daily life, humans are exposed to dust pollutants through different routes: dermal, ingestion, and inhalation. HI is as follows (Eq. 8):

8 $$\it {\text{HI}} = { }\mathop \sum \limits_{{{\it{i}} = {\text{1}}}}^{3} {\text{HQ}}_{{\it{i}}} = ({\text{ADD}}/{\text{RfD}})_{ing} + ({\text{ADD}}/{\text{RfD}})_{inh} + ({\text{ADD}}/{\text{RfD}})_{derm}$$

where ADD_ing_, ADD_inh_, and ADD_derm_ are the average daily dose (mg/kg day) for ingestion, inhalation, and dermal contact, respectively. The RfD is the maximum intake according to body weight per unit time that does not cause adverse reactions in the human body. If HI < 1, there is no significant risk of non-carcinogenic effects whereas for HI > 1, there is a chance that non-carcinogenic effects occur^20^. ADD formulas and RfD values are mentioned in Table S4, and Table S5.

#### Carcinogenic risk

The carcinogenic risk (CR) is the summation of the lifetime average daily dose (LADD_i_, mg/kg.day) for each exposure route multiplied by the cancer slope factor (SF_i_). LADD formula and SF values of PTMs analyzed in this study are presented in Table S5. The CR is calculated using the following equation (Eq. 9).

9 $$CR = \mathop \sum \limits_{i = 1}^{3} LADD_{i} \times SF_{i}$$

If CR < 1 × 10^−6^, there is a negligible carcinogenic risk while CR > 1 × 10^−4^ means the risk is unacceptable. CR between the range of 1 × 10^−6^ and 1 × 10^−4^ indicates acceptable or tolerable carcinogenic risk^21^.

## Results and discussion

### PTM concentrations

In Table 1, the descriptive statistics of PTMs in 50 dust samples from Qom city are described. The background values were used based on the concentrations of metals in the Upper Continental Crust. The mean concentration of As, Cd, Cu, Mo, Pb, Sb, and, Zn exceeded the background value. Also, Cd, Cu, Mo, Pb, Sb, and, Zn had a coefficient of variation (C.V.) greater than 50%, indicating a severe variability in PTMs concentrations in the atmospheric dust of the studied area^2^. Metals with C.V. < 50% (including Fe, Mn, Ni, Cr, Co, and As) might be from geogenic sources^22^.

**Table 1:** Statistical summary of total PTMs in windowsill dust samples of the studied area, mg/kg (n = 50).

Variable	Mean	Median	Min	Max	SD	C.V.%	UCC
As	16.83	15.10	10.10	33.20	5.86	34.83	1.5
Cd	0.80	0.33	0.25	4.80	1.17	146.08	0.098
Co	13.76	11.50	9.00	26.00	5.00	36.37	17
Cr	80.16	64.00	44.00	200.00	38.30	47.78	85
Cu	130.02	105.00	42.00	376.00	75.69	58.21	25
Fe	39,768.12	35,375.00	27,578.00	64,137.00	10,870.49	27.33	35,000
Mn	706.24	680.00	553.00	1455.00	134.52	19.05	600
Mo	2.74	2.30	1.20	6.50	1.52	55.47	1.5
Ni	39.96	35.00	22.00	83.00	12.79	31.99	50
Pb	367.64	145.50	54.00	1686.00	438.58	119.29	16
Sb	6.43	1.02	0.83	50.00	10.45	162.57	0.2
Zn	708.66	500.00	165.00	2478.00	581.64	82.08	71

Table 2 shows the comparison of the average concentration of PTMs in this study (determined in four land-use categories) with those reported in Iran cities and other countries. Industrial samples contained the highest amount of all PTMs—except for Mn and Cu. PTMs including As, Sb, Pb, and Zn were considerably higher than the background values in all the functional sectors.

**Table 2:** Summary of the average PTMs concentration (mg/kg) in dust from different cities in the national and international scales.

City	Region	Elements												Dust type	Dust size (µm)	Reference
		As	Cd	Co	Cr	Cu	Fe	Mn	Mo	Ni	Pb	Sb	Zn			
UCC		1.5	0.098	17	85	25	35,000	600	1.5	50	16	0.2	71			^18^
Qom	RSD^a^	14.8	0.3	10.9	57.7	136.4	33,414	703	1.9	33.2	102.8	1.9	429.3	Windowsill dust	< 63	Present study
	CMR^b^	14.5	0.4	10.7	73.0	159.1	33,756	673	2.5	42.1	219.0	4.0	634.6			
	GRN^c^	12.4	0.3	10.6	65.2	51.0	37,183	784	1.3	30.2	113.4	0.9	223.2			
	IND^d^	22.5	1.8	20.7	120.0	128.5	53,110	707	4.5	50.8	904.6	15.9	1292.0			
	Total	16.8	0.8	13.8	80.2	130.0	39,768	706	2.7	40.0	367.6	6.4	708.7			
**National studies**																
Tehran	Total	7.5	5.7	–	60.9	266.5	43,498	1012	5.0	45.2	240.1	11.0	789.3	Street dust	< 63	^23–25^
Abadan	Total	7.0	0.5	7.5	50.0	113.0	–	–	–	57.0	59.0	–	288.0	Street dust	< 63	^26^
Bandar Abbas	RSD, CMR	10.0	0.5	9.8	70.6	166.3	27,607	484	–	67.5	92.3	3.8	268.7	Road dust	< 63	^11,27^
Mashhad	Total	–	0.3	10.6	85.6	112.8	25,109	480	2.9	–	100.5	3.2	325.8	Road dust	< 63	^28^
Yazd	Total	12.1	0.4	9.2	91.4	90.3	26,800	500	2.2	45.7	79.6	2.7	411.4	Street dust	< 63	^29^
Isfahan	Total	22.1	2.1	13.9	82.1	182.3	–	–	–	66.6	393.3	6.9	707.2	Road dust	< 63	^9^
Dezful	RSD, IND	–	–	11.7	35.7	54.0	11,280	365	–	79.0	54.0	–	170.0	Street dust	< 63	^30^
Bushehr	Total	6.4	0.3	5.1	45.8	118.0	16,716	346	6.6	35.0	94.9	3.3	283.0	Street dust	< 63	^31^
Shiraz	RSD	6.6	0.5	–	67.2	136.3	20,254	438	–	77.5	115.7	4.8	403.5	Street dust	< 63	^32^
**International studies**																
China (Shijiazhuang)	Total	15.5	–	36.5	59.7	43.9	–	540	–	24.5	42.1	–	238.8	Road dust	< 63	^33^
China (Anyang)	IND	104.5	5.3	15.8	143.3	86.9	–	1475	–	28.1	228.9	–	672.6	Windowsill dust	< 60	^2^
China (Jiyuan)	IND	–	174	–	–	466.8	–	–	–	–	5420	–	1837	Windowsill dust	45–125	^34^
India (Dehradun)	Total	4.4	–	–	46.7	80.4	12,509	316	–	35.4	138.8	–	217.1	Road dust	< 63	^35^
India (Sonbhadra)	IND	6.2	0.15	–	51.0	39.5	–	394	–	24.7	22.2	–	109.7	Road dust	< 63	^36^
Bangladesh (Dhaka)	Total	3.6	–	8.5	65.8	–	25,150	554	–	–	–	0.7	122.0	Road dust	< 63	^37^
Bangladesh (Gazipur)	Total	2.1	9.1	–	83.3	44.9	–	–	–	33.5	40.9	–	239.1	Street dust	< 63	^38^
Saudi Arabia (Riadh)	IND	–	0.1	4.5	29.7	24.0	12,900	210	–	26.7	16.1	–	599.0	Ground dust	< 63	^39^
Spain (Madrid)	RSD, CMR	–	1.25	–	100.0	411	–	–	–	42.0	290.0	–	895.0	Street dust	< 50	^40^
Korea (Busan)	Total	17.2	4.1	21.6	531	559	–	–	–	139	385	31.6	2511.0	Road dust	< 63	^41^

^a^Residential, ^b^commercial, ^c^greenspace, ^d^industrial.

Most of the studies have sampled dust from the street and roads. For this study, we used the windowsill dust at a height of 1.5 m. According to Table 2, dust contamination in Qom city is significant and as high as Tehran and Isfahan metropolises. Cobalt in Qom dust recorded the highest concentration among the other cities—same as the Isfahan—despite being lower than the background value. Manganese, Fe, and Zn in Qom dust were higher than in the other cities in Iran, except for Tehran (Iran). Lead concentration in Qom dust was almost similar to the value reported from Isfahan (Iran) and higher than the average values obtained in other cities of Iran (Table 2). Antimony had a significant amount in the industrial sector, and its average overall concentration is placed after Tehran and Isfahan. To sum up, Qom can be listed as the top three cities in Iran, along with Tehran and Isfahan in terms of dust pollution.

On the international scale, As and Pb concentrations in Qom were almost similar to Busan (Korea). Arsenic was significantly higher than the estimations from the other countries mentioned in Table 2, except Anyang (China). The concentrations of Pb, Cu, and Zn were found lower compared to Jiyuan (China) industrial city. The concentration of Zn was lower than Jiyuan (China), Madrid (Spain), and Busan (Korea), but higher than the other listed countries. Notably, Fe and Mn contents in this study were almost higher than in the other cities (except Tehran, Iran).

### PTM pollution assessment

**Figure 2:**
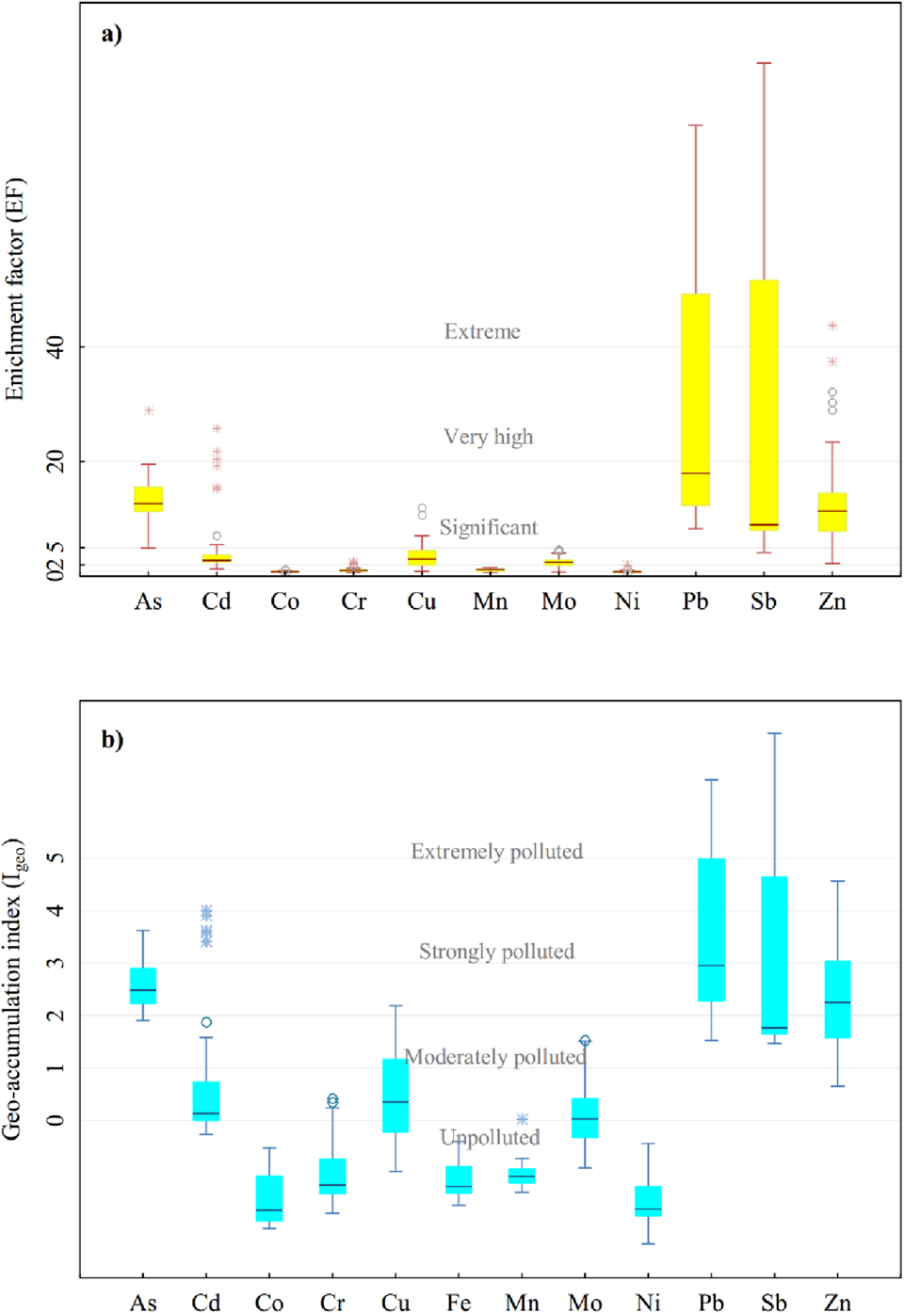
Box plot of the (**a**) enrichment factor (EF), and (**b**) geo accumulation index (I_geo_) for the dust samples in the studied area.

The EF and I_geo_ values of PTMs in Qom’s dust are given in Fig. 2 and the details are presented in Table S6. The mean EFs were found in the order of Sb > Pb > Zn > As > Cd > Cu > Mo > Cr > Mn > Ni = Co. Antimony (38.55) and Pb (35.13) had the highest average EF values, which means they were enriched very high in the windowsill dust. Also, they had a wide range of EF values in the 50 stations: from 4.0 to 227.0 for Sb, and from 8.3 to 140.8 for Pb which might reflect the existence of discrete multiple sources in the studied area. The degree of enrichment for Pb and Sb in the industrial sector was extreme and in the commercial sector was very high; also, the other sectors were significantly enriched. Zinc and As had a more homogenous enrichment in the area. In all the functional sectors, 95% and 84% of stations were significantly enriched by As and Zn, respectively. Copper, Cd, and Mo were moderately enriched in all functional sectors, but the greenspace sector had minimal enrichment by these elements. Some areas in the industrial sector had significant to very high enrichment of Cd. The EF value indicated Co, Cr, Mn, and Ni were minimally enriched in all the stations.

The highest average values of I_geo_ were obtained in the order of Pb > Sb > As > Zn. PTMs included Co, Cr, Ni, Fe, and Mn were categorized as unpolluted and Cd, Cu, and Mo were in the category of unpolluted to moderately polluted. In the industrial zone, the windowsill dust was extremely polluted with Sb and Pb. The sequence of contamination intensity with Pb, Zn and Sb according to land use was: industrial > commercial > residential > greenspace. The highest concentration of arsenic in the study area belongs to the industrial area.

**Figure 3:**
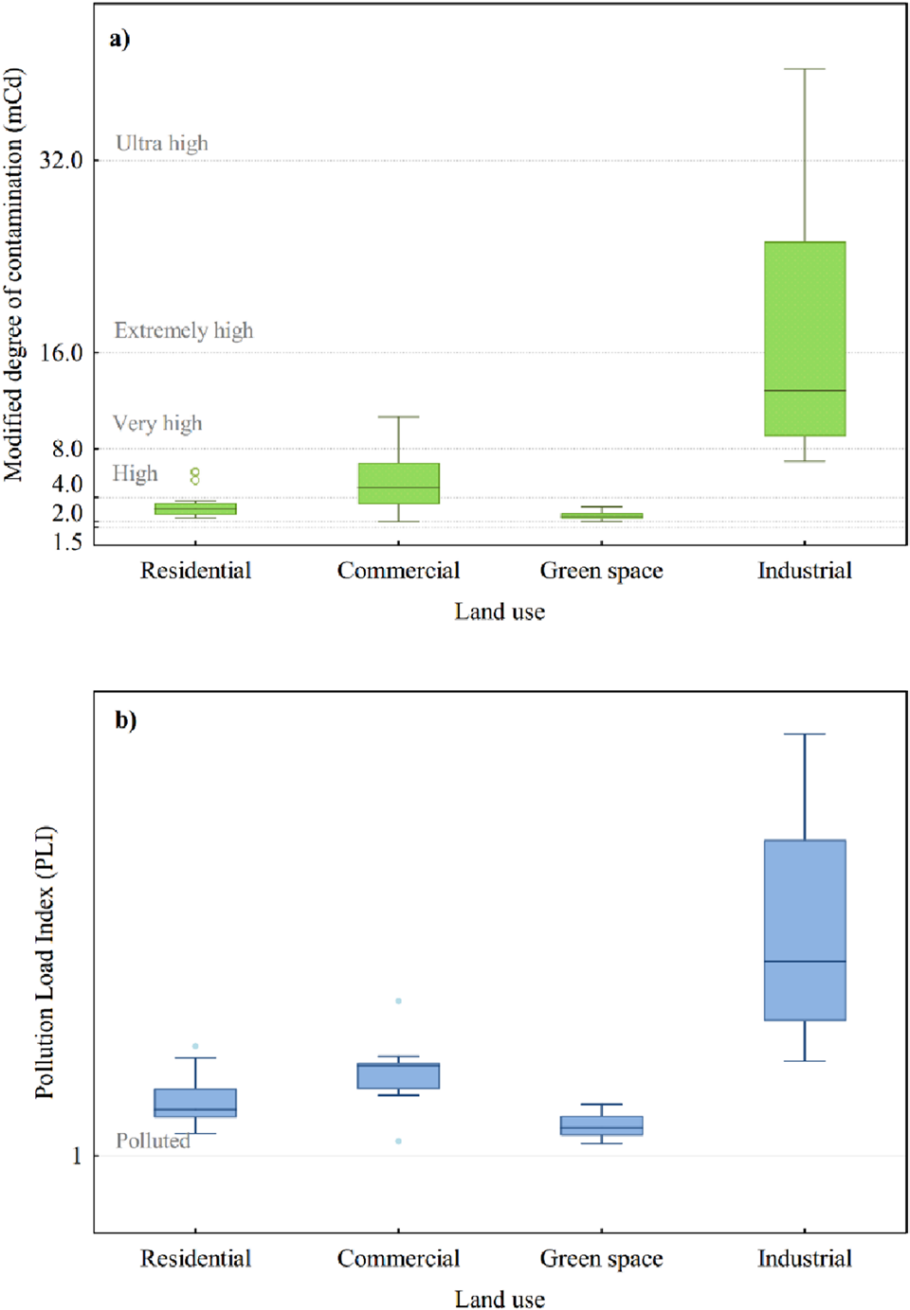
Pollution level indexes (**a**) mC_d_ and (**b**) PLI, based on four functional areas.

To evaluate the pollution level based on land use, PLI and mC_d_ indices were utilized (Fig. 3). These cumulative indices showed that the dust in Qom city is considerably contaminated with PTMs. According to the PLI index, all the stations were categorized as polluted sites. The PLI_zone_ values were in the order of industrial (3.77) > commercial (2.05) > residential (1.67) > green space (1.38). This pattern was also repeated with the mC_d_ index. The mC_d_ for the industrial sector ranged from 6.98 (high contamination level) to 39.60 (ultra-high contamination level). In the commercial sector, fifty percent of dust samples were classified as having a high degree of contamination. All the greenspace stations were in the moderate pollution category. This shows the possible effect of tree density in diminishing the risk of dust pollution to the receptors.

### Spatial distribution of PTMs

**Figure 4:**
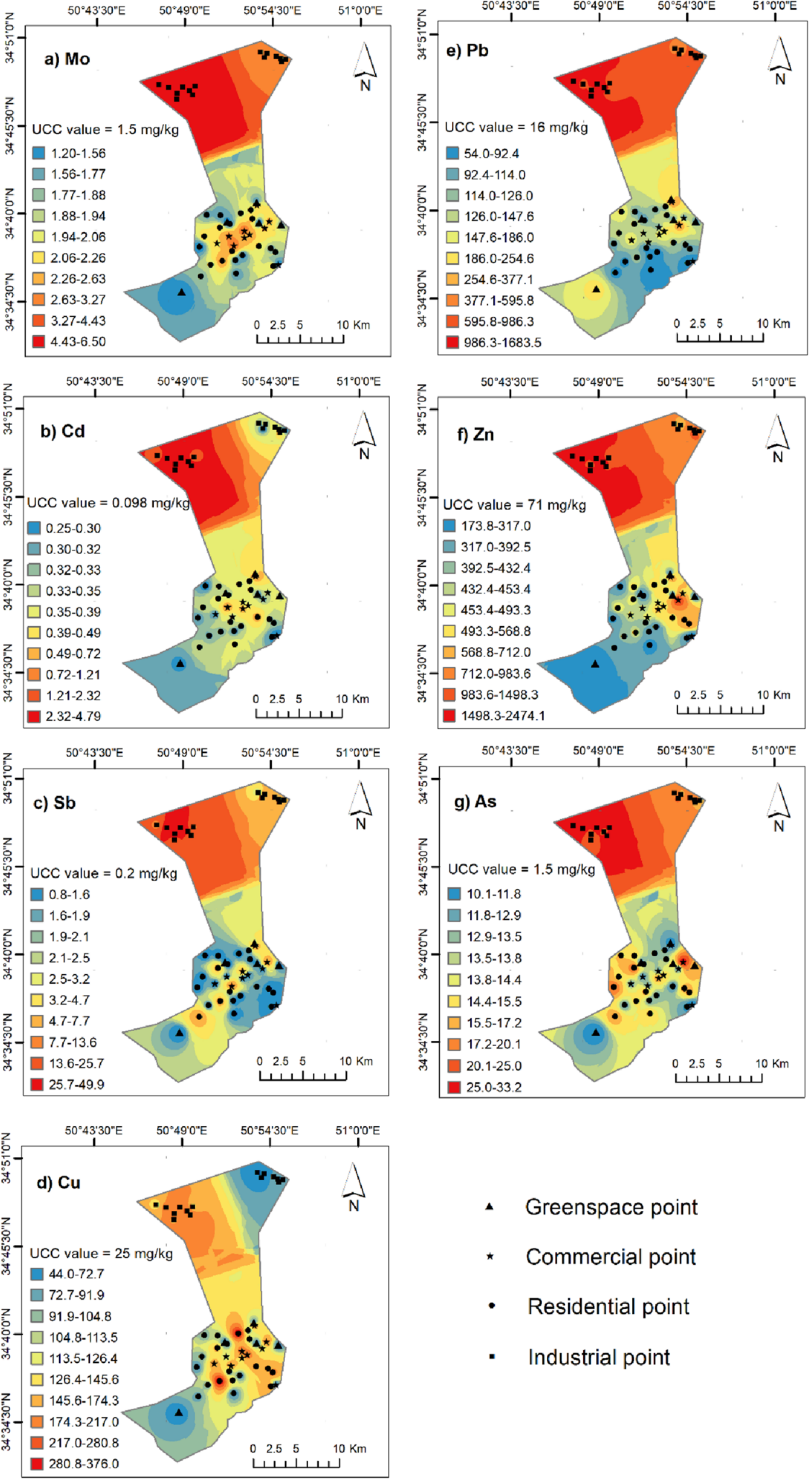
Spatial distribution maps of seven PTMs in windowsill dusts of Qom, Iran. This map was constructed using ArcGIS version 10.3. (https://www.esri.com/en-us/arcgis/products/arcgisdesktop/overview).

The As, Cd, Cu, Sb, Pb, and Zn content in 100% of the dust samples exceeded the background value. Spatial distribution maps were generated for the hotspot PTMs (As, Sb, Pb, Cd, Cu, Mo and Zn) by applying the inverse distance weighted (IDW) interpolation method (ArcGIS 10.3). Figure 4 demonstrates that PTMs dispersions were slightly influenced by the prevailing wind direction (from the west), suggesting they came from the point- or area- sources. On the other hand, the K–S test showed that the overall distribution of PTMs was not normal in the studied region. This might signify the influence of industrial activities and the presence of multiple sources of dust.

The highest pollution load of PTMs belonged to the industrial section. The level of pollution gradually decreased from Shokouhieh to Mahmoudabad industrial zones. The reason is related to more active industries, a closed environment, and more construction existing in Shokouhieh industrial town than in Mahmoudabad industrial town.

There is a clear decreasing trend from the central part to southern (downtown area) and southwestern (suburb area) parts of the city. In fact, these parts are diffusely populated and the southwestern part is almost new with lots of barren lands. Copper, Mo, and Cd show high concentrations toward the central part of the city. Educational, cultural and commercial activities are mainly located in the central part of the city. Also, historical and religious districts in the city center are accompanied by a huge influx of tourists throughout the year. For this reason, the central part of the city has various public transportations such as bus stands and taxi stations, and is dominated by a high load of motorcycles.

In the eastern part of the city, some hotspots can be observed (Fig. 4). This part includes an important transportation system (like highways and a complex interchange) where exhaust traffic emissions might be a probable source of As, Sb, Pb, and Zn. Unlike Pb and Zn, several peaks of As are scattered in the western part, suggesting an area source might exist in the region. It is noteworthy that the western area is densely populated with lots of residential buildings. Bisht et al. (2022)^35^ also observed hotspots of As in the residential area of Dehradun, India.

### PTM potential sources

**Figure 5:**
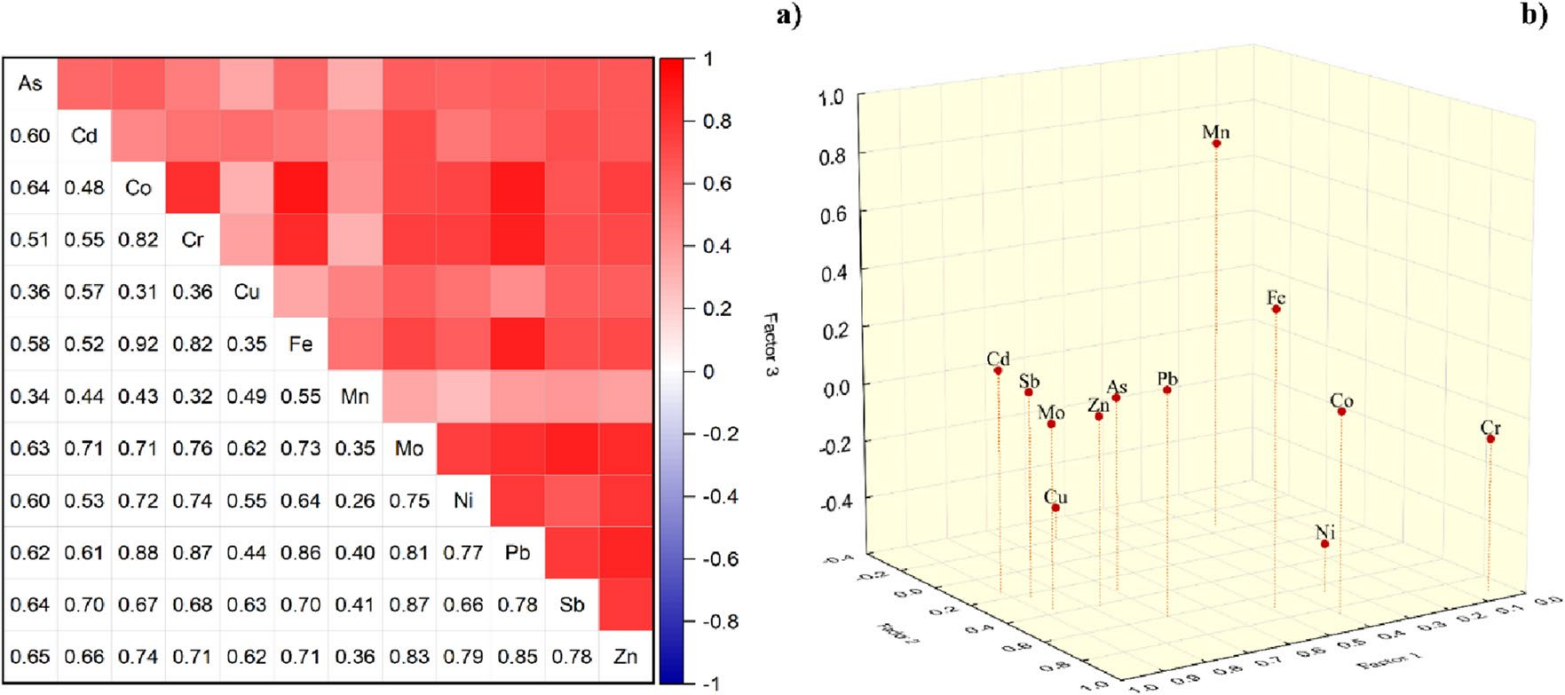
(**a**) Spearman correlation matrix, (**b**) principal component analysis (PCA) diagrams.

To evaluate the relationship between PTMs in dust samples, the Spearman correlation and PCA were developed (Fig. 5) and more details are given in Table S7. Statistical analysis can help to identify the potential source of contamination in urban dust. The Spearman correlation was significant at *p* < 0.05, and those correlations higher than 0.8 show a very strong correlation. The Kaiser–Meyer–Olkin (KMO) was 0.784 and Bartlett’s sphericity test was less than 0.05, which showed the reliability of PCA data. Three components with eigenvalues greater than 1.0, account for 77.9% of the total variances.

*First component (PCA1)* of PCA (55.40% loading) includes Cd (0.9), Mo (0.87), Sb (0.87), Zn (0.78), Pb (0.7), As (0.69), and Cu (0.6). The highest correlation was determined for Sb-Mo (0.87) and Mo-Zn (0.83) and Mo-Pb (0.81). In this study, more than 90% of these PTM values were higher than the background values in all the functional areas. The concentration of PTMs implies that the major part of this group originates from anthropogenic sources. However, the interpolated spatial distributions and Spearman analysis suggest that they might not stem from the same source. Traffic-related materials in the central and eastern parts of the city and industrial emissions in the northern region predominated over the other urban activities (like household activities and construction) have less contribution.

According to the pollution indices, the windowsill dust was enriched with Pb, Zn, Sb, and Cd. Metals such as As, Sb, Cd, Zn, and Pb are released from non-ferrous/ferrous alloy plants located in Mahmoudabad and Shokouhieh industrial zones. Arsenic also can stem from Cu-smelting slags around the city. Khorasanipour and Esmaeilzadeh^42^ stated that As in the topsoil came from Sarcheshmeh Cu-smelting slags via wind-blowing particles. Arsenic pollution in dust can also be attributed to household activities (like waste disposal and thermal system)^43^ and construction work^44^ (like paints, cement usage and demolition). Apart from smelting emissions, Sb can be derived from products like plastics and polyethylene terephthalate (PET), and textiles^45^, all of which exist in the industrial section of the studied area. It is noteworthy that in today’s world, traffic pollution is an inevitable consequence of urbanization. Many previous studies also confirmed the presence of Pb, Zn, Cd, As, and Sb in the street dust of high traffic nodes^2,9,11^. Lead, Zn, and As are correlated with motor vehicle exhausts and are widely used in automobile parts^33^. Copper, Mo, Cd, and Sb are utilized in lubricating oils and automotive greases.

*The second component (PCA2)* involves Cr, Co, Fe, and Ni accounting for 13.46% of the variance. PCA 2 loaded heavily on Cr (0.92) and Co (0.86), with moderate loading on Ni (0.66) and Fe (0.70). Cobalt showed a remarkable positive correlation with Cr (0.82) and Fe (0.92), which reflects they stemmed from the same source. It is noteworthy that all the elements in this component were lower than the background value and had an EF < 1.5 which suggests the natural originality of the elements^11^. Generally, a substantial proportion of Ni, Fe, Cr and Co comes from lithogenic materials deposited as dust in the atmosphere. However, 36% of Fe and 28% of Cr exceeded the background value. The correlation of Cr and Fe with Pb were respectively 0.87 and 0.86, indicating smelting activities as the potential source of Fe and Cr^7^. An iron/steel smelting plant is located in the Mahmoudabad industrial zone (Fig. 1) which could be the reason for increasing Fe and Cr concentrations in the windowsill dust of the industrial section. Consequently, Fe and Cr arise from a mixture of natural and anthropogenic sources (especially in the industrial section). Long et al.^7^ also reported Fe and Cr in the same PCA component with higher concentrations in the steel plant area compared to other functional areas in Panzhihua city, China.

*The third component (PCA3)* with a total variance of 10.06% included Mn (0.76). Mn had a weak to moderate correlation with the other studied PTMs. The manganese content in 95% of the samples exceeded the UCC value. Manganese had the lowest CV which shows this element was normally distributed in the studied area. Particularly, one station in a park had an extreme value that might be due to an accidental event; thus it was considered as an abnormal value. Fard et al.^46^ also reported an approximately stable seasonal variation of Mn in the urban region of Qom. Venarj Mn mine, the largest Mn mine in Iran (1962–present), distances about 30 km from Qom city. The mining activities and related transportation disperse high amounts of Mn minerals into the atmosphere. Thus, PCA 3 might refer to the mine-associated sources in windowsill dust.

To sum up, finding the origination of PTMs in the dust is a laborious task, especially for contaminants with low concentrations. Descriptive statistics revealed three main routes of contamination in the windowsill dust: (1) a mixture of anthropogenic sources including industrial activities (alloys, chemical, textile and plastic productions) and urban activities (like traffic emissions—vehicle exhaust, bake and tire wear); (2) lithogenic/geogenic sources; and (3) mining activities (processing, transportation and waste).

### Risk assessment

#### Ecological risk assessment

**Figure 6:**
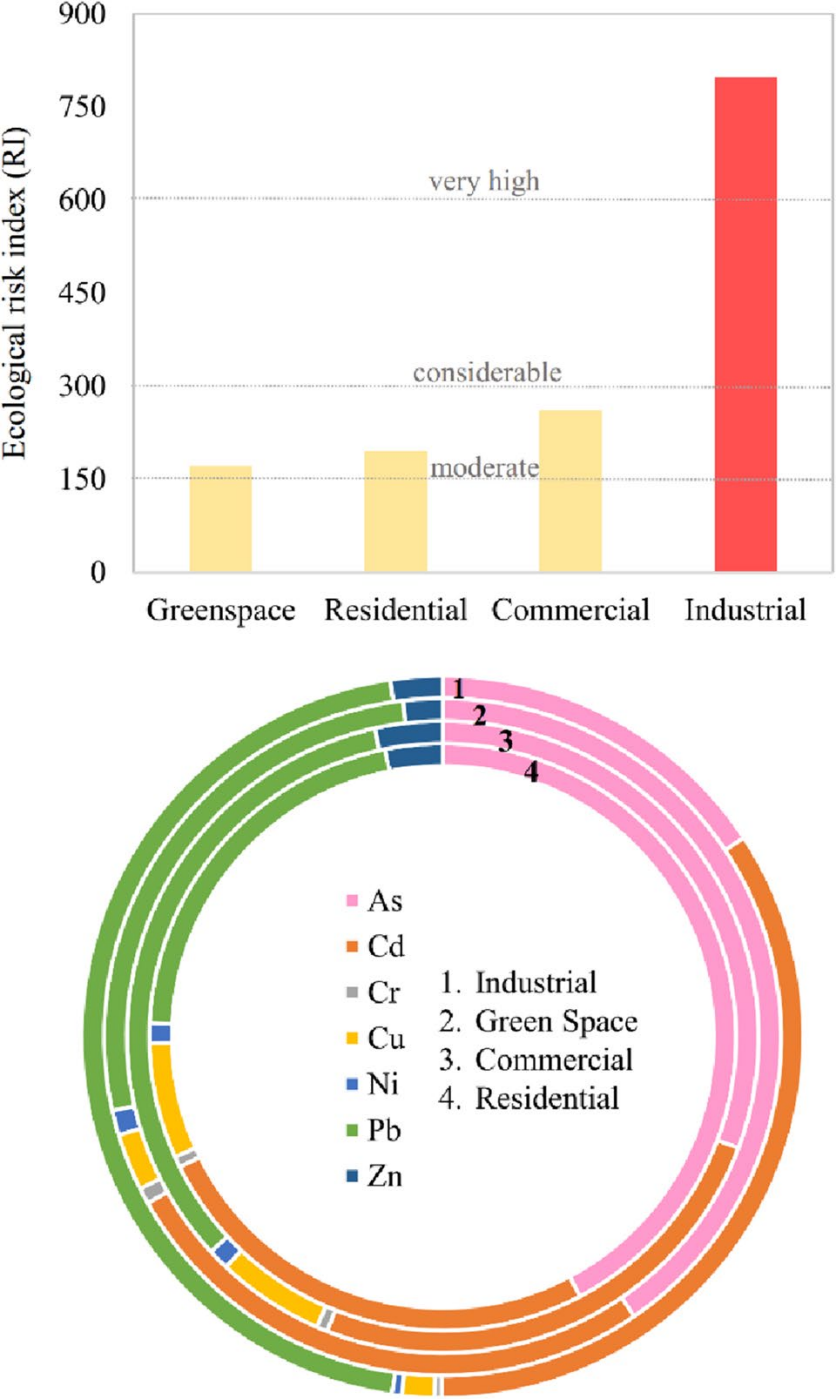
Potential ecological risk index and donut contribution of metals regarding functional areas.

The average PERI values indicated that the samples from the industrial zone can be categorized as very high ecological risks (Fig. 6). PTMs in samples of all residential sectors and most greenspaces and commercial sectors had moderate ecological risk (about 62% of all samples). However, two points in the greenspace sector and the commercial sector and two points in commercial sectors demonstrated low ecological risk and considerable ecological risk, respectively; all of the four stations were located in the east of Qom.

In residential and greenspace sections, As had the highest contribution followed by Cd and Pb. While in commercial and industrial sections the highest contributions in RI belonged to Pb. Among the elements considered in RI, Cd had a low concentration with a remarkable contribution. Cd has the maximum T_r_ (30) which is related to its high half-life time of about 25–30 years^47^. RI index was first introduced by Hakanson in 1980, and the contribution of modern contaminants like Sb has not been considered in the index. However, studies in the last two decades affirm the bioaccumulation and toxicological effects of Sb on humans^48^ and the ecosystem^49^.

#### Health risk assessment

**Figure 7:**
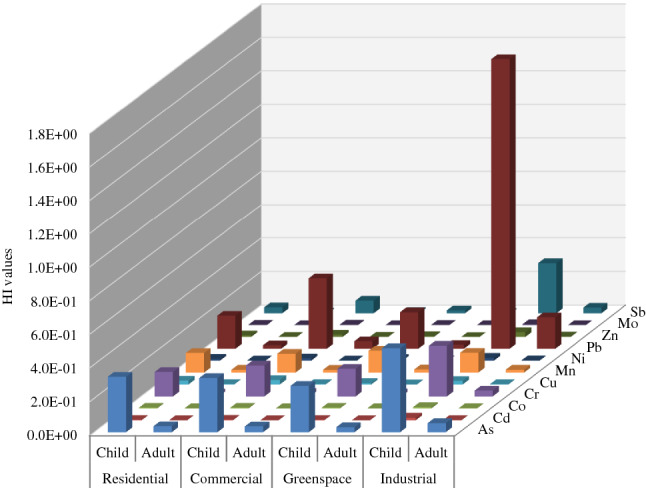
Non-carcinogenic health risk of PTMs for children and adults based on functional areas.

PTMs in dust particles could cause health issues for local residents, especially sensitive individuals and children^50^. The non-carcinogenic health risk of PTMs (HIs) through three possible pathways (oral ingestion, dermal, and inhalation) was measured for children and adults based on functional areas (Fig. 7, Table S8).

The average HI values of all PTMs regarding land-use type decreased in the order of industrial > commercial > residential ≈ greenspace. The five PTMs with the highest overall HI are ranked as follows: Pb > As > Cr > Mn > Sb (Fig. 7). The HI values in all the sections were lower than the permissible level (1.00), except for Pb. In the industrial section, Pb recorded the highest HI value for children (HI = 1.73) which exceeded the acceptable value. The HI values were 10 times higher for children than adults indicating they are more susceptible to PTMs in the dust.

The dominant pathway for noncancerous risk was ingestion followed by dermal contact and inhalation. The trend is in line with previous research^25,51,52^. However, for Co and Mn, the descending order was different as follows ingestion > inhalation > dermal contact. The highest contribution of HQ_inh_ and HQ_derm_ to HI was measured for Co (34.0%) and Cd (29.0%), respectively.

**Figure 8:**
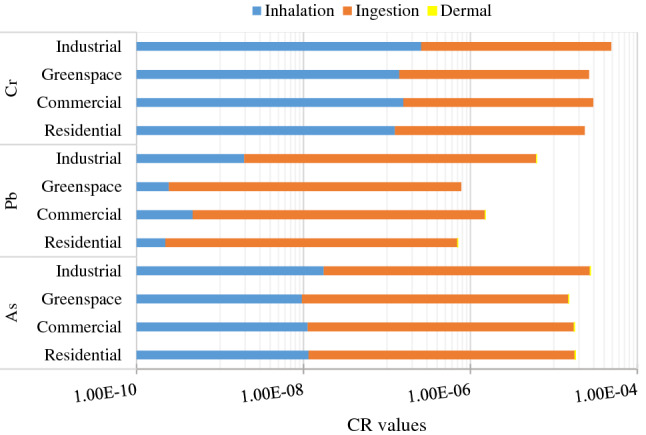
Carcinogenic risk of Cr, As and Pb through different pathways (inhalation, ingestion, dermal).

In this study, the carcinogenic risk from windowsill dust was estimated for the carcinogens including Cd, Co, Cr and Ni, Pb, and As through the possible routes (Fig. 8, Table S9). The contribution of PTMs to CR decreased in the order of Cr (3.24E−05) > As (2.05E−05) > Pb (2.52E−06) > Co (6.91E−09) > Ni (1.72E−09) > Cd (2.58E−10). The average CR values for target PTMs through inhalation ranged from 7.9E−10 to 1.7E−07, which remained in the safety zone (CR < 1 × 10^−6^). The highest amount of CR belonged to Cr (4.9E−05) in the industrial zone, while pollution assessments identify the level of Cr pollution as uncontaminated in the windowsill dust. The high CR value of Cr mainly is related to the high slope factor (42 kg day/mg). Therefore, this is difficult to ascertain Cr cancerous effect considering its low speciation in the studied dust. Several studies on dust pollution have reported Cr as the highest contributor to carcinogenic effects^4,7,53^. Rehman et al.^50^ indicated that despite a moderate level of pollution, Cr in the indoor dust had a high CR close to the unacceptable range. Taiwo et al.^4^ also identified Cr as the highest contributor of CR in Nigeria road dust, while its concentration was lower than the background value.

The contribution of Pb and As to CR values in the windowsill dust regarding carcinogen routes was ingestion > inhalation > dermal (Fig. 8). While the contribution of Cr to carcinogenic risk was higher through inhalation than ingestion. The reports concluded that the primary exposure route of Cr is inhalation^54^. Considering the predominant forms of Cr in the environment, Cr^VI^ is more toxic than Cr^III^. Exposure to Cr^VI^ can cause immunological diseases, dental effects and carcinogenic effects (lung cancer, nose and nasal sinus cancer, suspected laryngeal and stomach cancers)^54,55^.

The result of health risk from target PTMs in windowsills of Qom indicates significant chronic exposure to Pb can take place for children in the industrial zone. The ingestion route is the most probable pathway for children due to their hand–to–mouth behavior^56^. Lead can bio-accumulate in the body without any obvious symptoms of toxicity^56^. The total CR values for Pb, Cr and As in different land-use types were in the range of tolerable carcinogenic risk (1 × 10^−4^ < CR < 1 × 10^−6^), except for Pb in the greenspace and residential sections (CR < 1 × 10^−6^). Lung cancer and skin cancer show a clear correlation with As exposure through oral and inhalation routes^57^. Moreover, Long-time exposure to As increases the risk of skin lesions and Blackfoot disease^57^. Since urbanization and industrialization continue in Qom city, the carcinogenic risk of As, Pb, and Cr in the dust may exceed the safe level in the future. Furthermore, the present study only identifies possible exposure to PTMs via street dust, while exposure from other sources (like food, indoor dust, and soil) would aggravate the exposure and carcinogenic risks. United States Environmental Protection Agency (USEPA) has not yet included Sb in the category of carcinogen elements. However, reports confirmed the possible carcinogenic effects of Sb on animals^58^ and humans due to long-term exposure^48^.

### limitations and uncertainties

Several limitations and uncertainties still exist in the present study. The concentration of HMs in dust may be influenced by meteorological conditions (such as rainwater and dust storm). Nevertheless, seasonal variations were not taken into account. Sampling was done from a height of 1–2 m above the ground. In order to investigate the effect of height on the concentration of HMs in dust, it is suggested to take samples from low-, medium-, and high-rise buildings. The possible concentration difference in various particle sizes is not considered. This study did not determine the bioavailability of metals in dust, an easy transport indicator in the food chain. The average upper continental crust (UCC) values were used as the background reference contents in this study due to the lack of background surface dust values. Other routes of exposure to HMs (contaminated soil, food, and water) and the presence of organic pollutants (e.g., microplastics and polycyclic aromatic hydrocarbons) have not been evaluated in the studied dust, although they would elevate the aggregation risk. Uncertainty is inherent in human health risk assessment even when the most accurate data and the most sophisticated models are used. Given the significant uncertainties associated with the estimates of toxicity values, exposure factors, and the absence of site-specific biometric factors, these results should be regarded as screening data. Further research should be undertaken before conclusions regarding potential health effects are drawn.

## Conclusion

The assessment and monitoring of PTMs in topsoil and surface dust are essential for environmental management decisions. A comprehensive study was conducted on dust pollution collected from windowsills in Qom (Iran) based on land-use types (residential, industrial, commercial, and greenspace). The results of pollution, ecological risk, and health risk assessment as well as source apportionment for the target PTMs (As, Cd, Co, Cr, Cu, Fe, Mn, Mo, Ni, Pb, Sb, and Zn) revealed that:

- The highest concentration of target PTMs was found in the industrial sector, and the lowest concentration was recorded in the greenspace sector. Also, the mean concentration of As, Cd, Cu, Mo, Pb, Sb, and Zn exceeded the background values.
- Antimony, Pb, As, and Zn had the highest geo-accumulation index. The windowsill dust was very highly enriched in Sb and Pb. According to accumulative indices, urban dust was categorized as polluted with high levels of contamination in the order of industrial > commercial > residential > greenspace.
- Industrial activities (like none/ferrous alloy production and smelting) and traffic (like vehicle exhausts and lubricants) have substantial roles to play polluting urban dust with As, Pb, Sb, Cu, and Zn. At the next level, mining activities and other urban activities (like household activities and construction) influenced the quality of deposited dust. Industrial activities have left a high ecological footprint on windowsill dust.
- The predominant routes of health risks contribute to ingestion. The non-carcinogenic risk of Pb in the industrial zone was undesirable for children. The carcinogenic risk of Cr, Pb and As were in a tolerable range.

Evaluation of urban dust, as an indicator of environmental quality, showed the effect of unsustainable development in Qom city. Qom dust pollution is comparable to the other industrial and megacities like Tehran (Iran) and Busan (Korea). An environmental management plan is required for monitoring the situation, finding the exact sources, and informing the residents. Planting trees, considering restriction regulations for industrial emissions, and optimizing environmental policies for mining activities and traffic emissions (like using unleaded fuels and technical car inspections) are among the effective strategies for dealing with dust contamination.

## Supplementary Information

Supplementary Information.

## Data Availability

The datasets used and/or analyzed during the current study are available from the corresponding author on reasonable request.

## References

[1] Pilehvar AA (2021). Spatial-geographical analysis of urbanization in Iran. Humanit. Soc. Sci. Commun..

[2] Han Q (2021). Pollution effect assessment of industrial activities on potentially toxic metal distribution in windowsill dust and surface soil in central China. Sci. Total Environ..

[3] Tarafdar A, Sinha A (2019). Health risk assessment and source study of PAHs from roadside soil dust of a heavy mining area in India. Arch. Environ. Occup. Health.

[4] Taiwo A (2020). Spatial distribution, pollution index, receptor modelling and health risk assessment of metals in road dust from Lagos metropolis, Southwestern Nigeria. Environ. Adv..

[5] Chau B, Witten ML, Cromey D, Chen Y, Lantz RC (2021). Lung developmental is altered after inhalation exposure to various concentrations of calcium arsenate. Toxicol. Appl. Pharmacol..

[6] Ferreira-Baptista L, De Miguel E (2005). Geochemistry and risk assessment of street dust in Luanda, Angola: A tropical urban environment. Atmos. Environ..

[7] Long Z (2021). Contamination, sources and health risk of heavy metals in soil and dust from different functional areas in an industrial city of Panzhihua City, Southwest China. J. Hazard. Mater..

[8] Tian S, Liang T, Li K (2019). Fine road dust contamination in a mining area presents a likely air pollution hotspot and threat to human health. Environ. Int..

[9] Soltani N (2015). Ecological and human health hazards of heavy metals and polycyclic aromatic hydrocarbons (PAHs) in road dust of Isfahan metropolis, Iran. Sci. Total Environ..

[10] Shahab A (2020). Pollution characteristics and toxicity of potentially toxic elements in road dust of a tourist city, Guilin, China: Ecological and health risk assessment. Environ. Pollut..

[11] Heidari M, Darijani T, Alipour V (2021). Heavy metal pollution of road dust in a city and its highly polluted suburb; quantitative source apportionment and source-specific ecological and health risk assessment. Chemosphere.

[12] Dytłow S, Górka-Kostrubiec B (2021). Concentration of heavy metals in street dust: An implication of using different geochemical background data in estimating the level of heavy metal pollution. Environ. Geochem. Health.

[13] Zhou H, Chun X, Lü C, He J, Du D (2020). Geochemical characteristics of rare earth elements in windowsill dust in Baotou, China: Influence of the smelting industry on levels and composition. Environ. Sci. Process. Impacts.

[14] Rezaei F, Saghafipour A, Mirheydari M, Eshagh Hosseini S (2018). Trend of cancer incidence in Qom province in a period of 8 years (2007–2014). J. Health.

[15] Mosammam HM, Nia JT, Khani H, Teymouri A, Kazemi M (2017). Monitoring land use change and measuring urban sprawl based on its spatial forms: The case of Qom city, Egypt. J. Remote Sens. Space Sci..

[16] Darabi H, Jafari A, Akhavan Farshchi K (2016). Climate change analysis and its’ impacts in Qom province, Iran. J. Environ. Sci. Stud..

[17] Asadzadeh S, de Souza Filho CR (2020). Characterization of microseepage-induced diagenetic changes in the Upper-Red Formation, Qom region, Iran. Part I: Outcrop, geochemical, and remote sensing studies. Mar. Pet. Geol..

[18] Taylor SR, McLennan SM (2001). Chemical composition and element distribution in the Earth’s crust. Encycl. Phys. Sci. Technol..

[19] Abrahim G, Parker R (2008). Assessment of heavy metal enrichment factors and the degree of contamination in marine sediments from Tamaki Estuary, Auckland, New Zealand. Environ. Monit. Assess..

[20] USEPA (2001). Risk Assessment Guidance for Superfund: Volume III-Part A, Process for Conducting Probabilistic Risk Assessment.

[21] USEPA (1989). Risk Assessment Guidance for Super Fund.

[22] Bineshpour M, Payandeh K, Nazarpour A, Sabzalipour S (2021). Status, source, human health risk assessment of potential toxic elements (PTEs), and Pb isotope characteristics in urban surface soil, case study: Arak city, Iran. Environ. Geochem. Health.

[23] Saeedi M, Li LY, Salmanzadeh M (2012). Heavy metals and polycyclic aromatic hydrocarbons: Pollution and ecological risk assessment in street dust of Tehran. J. Hazard. Mater..

[24] Dehghani S, Moore F, Keshavarzi B, Beverley AH (2017). Health risk implications of potentially toxic metals in street dust and surface soil of Tehran, Iran. Ecotoxicol. Environ. Saf..

[25] Mihankhah T, Saeedi M, Karbassi A (2020). A comparative study of elemental pollution and health risk assessment in urban dust of different land-uses in Tehran’s urban area. Chemosphere.

[26] Ghanavati N, Nazarpour A, Watts MJ (2019). Status, source, ecological and health risk assessment of toxic metals and polycyclic aromatic hydrocarbons (PAHs) in street dust of Abadan, Iran. Catena.

[27] Keshavarzi B (2018). Contamination level, source identification and risk assessment of potentially toxic elements (PTEs) and polycyclic aromatic hydrocarbons (PAHs) in street dust of an important commercial center in Iran. Environ. Manag..

[28] Najmeddin A, Moore F, Keshavarzi B, Sadegh Z (2018). Pollution, source apportionment and health risk of potentially toxic elements (PTEs) and polycyclic aromatic hydrocarbons (PAHs) in urban street dust of Mashhad, the second largest city of Iran. J. Geochem. Explor..

[29] Nematollahi MJ, Dehdaran S, Moore F, Keshavarzi B (2021). Potentially toxic elements and polycyclic aromatic hydrocarbons in street dust of Yazd, a central capital city in Iran: Contamination level, source identification, and ecological–health risk assessment. Environ. Geochem. Health.

[30] Sadeghdoust F, Ghanavati N, Nazarpour A, Babaenejad T, Watts MJ (2020). Hazard, ecological, and human health risk assessment of heavy metals in street dust in Dezful, Iran. Arab. J. Geosci..

[31] Abbasi S (2017). Investigation of microrubbers, microplastics and heavy metals in street dust: A study in Bushehr city, Iran. Environ. Earth Sci..

[32] Keshavarzi B, Tazarvi Z, Rajabzadeh MA, Najmeddin A (2015). Chemical speciation, human health risk assessment and pollution level of selected heavy metals in urban street dust of Shiraz, Iran. Atmos. Environ..

[33] Zuo L (2022). Concentrations, sources and ecological–health risks of potentially toxic elements in finer road dust from a megacity in north China. J. Clean. Prod..

[34] Xing W (2022). Metal contamination in soils and windowsill dusts: Implication of multiple sources on dust metal accumulation within a city affected by Pb smelting. Environ. Sci. Pollut. Res..

[35] Bisht L, Gupta V, Singh A, Gautam AS, Gautam S (2022). Heavy metal concentration and its distribution analysis in urban road dust: A case study from most populated city of Indian state of Uttarakhand. Spat. Spatio-temporal Epidemiol..

[36] Ahamad A (2021). Potentially toxic elements in soil and road dust around Sonbhadra industrial region, Uttar Pradesh, India: Source apportionment and health risk assessment. Environ. Res..

[37] Kormoker T (2022). Road dust–driven elemental distribution in megacity Dhaka, Bangladesh: Environmental, ecological, and human health risks assessment. Environ. Sci. Pollut. Res..

[38] Kabir M (2021). Potentially toxic elements in street dust from an urban city of a developing country: Ecological and probabilistic health risks assessment. Environ. Sci. Pollut. Res..

[39] Al-Swadi HA (2022). Sources, toxicity potential, and human health risk assessment of heavy metals-laden soil and dust of urban and suburban areas as affected by industrial and mining activities. Sci. Rep..

[40] Delgado-Iniesta MJ (2022). Estimation of ecological and human health risks posed by heavy metals in street dust of Madrid City (Spain). Int. J. Environ. Res. Public Health.

[41] Jeong H, Ra K (2022). Source apportionment and health risk assessment for potentially toxic elements in size-fractionated road dust in Busan Metropolitan City, Korea. Environ. Monit. Assess..

[42] Khorasanipour M, Esmaeilzadeh E (2016). Environmental characterization of Sarcheshmeh Cu-smelting slag, Kerman, Iran: Application of geochemistry, mineralogy and single extraction methods. J. Geochem. Explor..

[43] Ali N (2021). Arsenic and lead in the indoor residential settings of different socio-economic status; assessment of human health risk via dust exposure. Environ. Sci. Pollut. Res..

[44] Garelick H, Jones H, Dybowska A, Valsami-Jones E (2009). Arsenic pollution sources. Rev. Environ. Contam..

[45] Dousova B (2020). Environmental interaction of antimony and arsenic near busy traffic nodes. Sci. Total Environ..

[46] Fard RF, Naddafi K, Hassanvand MS, Khazaei M, Rahmani F (2018). Trends of metals enrichment in deposited particulate matter at semi-arid area of Iran. Environ. Sci. Pollut. Res..

[47] Genchi G, Sinicropi MS, Lauria G, Carocci A, Catalano A (2020). The effects of cadmium toxicity. Int. J. Environ. Res. Public Health.

[48] IRIS & EPA (2011). Integrated Risk Information System (IRIS) US Environmental Protection Agency.

[49] Casado M, Anawar H, Garcia-Sanchez A, Santa Regina I (2007). Antimony and arsenic uptake by plants in an abandoned mining area. Commun. Soil Sci. Plant Anal..

[50] Rehman A (2020). Characterizing pollution indices and children health risk assessment of potentially toxic metal (oid) s in school dust of Lahore, Pakistan. Ecotoxicol. Environ. Saf..

[51] Hou S (2019). Pollution characteristics, sources, and health risk assessment of human exposure to Cu, Zn, Cd and Pb pollution in urban street dust across China between 2009 and 2018. Environ. Intern..

[52] Li H-H (2017). Pollution characteristics and risk assessment of human exposure to oral bioaccessibility of heavy metals via urban street dusts from different functional areas in Chengdu, China. Sci. Total Environ..

[53] Yesilkanat CM, Kobya Y (2021). Spatial characteristics of ecological and health risks of toxic heavy metal pollution from road dust in the Black Sea coast of Turkey. Geoderma Reg..

[54] Den Braver-Sewradj SP (2021). Occupational exposure to hexavalent chromium. Part II. Hazard assessment of carcinogenic effects. Regul. Toxicol. Pharmacol..

[55] Hessel E (2020). Adverse Health Effects and Diseases Caused by Chromium-6: Updating the Scientific Literature and the Risk Assessment for Larynx Cancer in tROM.

[56] IRIS & USEPA. *Integrated Risk Information System (IRIS) US Environmental Protection Agency*. Chemical Assessment Summary National Center for Environmental Assessment. Lead and compounds (inorganic); CASRN 7439-92-1 (2004).

[57] IRIS & USEPA. *Integrated Risk Information System (IRIS) US Environmental Protection Agency*. Chemical Assessment Summary National Center for Environmental Assessment. Arsenic, inorganic; CASRN 7440-38-2 (2002).

[58] Saerens A, Ghosh M, Verdonck J, Godderis L (2019). Risk of cancer for workers exposed to antimony compounds: A systematic review. Int. J. Environ. Res. Public Health.

