# Supplementary material for: Health risk assessment and source apportionment of potentially toxic metal(loid)s in windowsill dust of a rapidly growing urban settlement, Iran
Source: Sci Rep. 2022 Nov 17;12:19736. doi: 10.1038/s41598-022-21242-z (PMC9672307; doi:10.1038/s41598-022-21242-z)
Supplement: Supplementary file 1 — Supplementary Information. [file 41598_2022_21242_MOESM1_ESM.docx]

**Table S1.** Method Detection Limits (MDL) (µg/l) of the studied elements for ICP-AES.

| As | Cd | Co | Cr | Cu | Fe | Mn | Mo | Ni | Pb | Sb | Zn |
| --- | --- | --- | --- | --- | --- | --- | --- | --- | --- | --- | --- |
| 3.7 | 0.2 | 0.6 | 0.5 | 0.5 | 0.5 | 0.1 | 1.2 | 0.9 | 1.9 | 3.4 | 0.3 |

**Table S2.** Certified and measured metal contents for MESS-3 (mg/kg).

| Metal | Certified value | Measured value | Recovery efficiency (%) |
| --- | --- | --- | --- |
| As | 21.2 | 20 | 94 |
| Cd | 0.24 | 0.25 | 104 |
| Co | 14.40 | 13.9 | 97 |
| Cr | 105 | 104 | 99 |
| Cu | 33.90 | 33.3 | 98 |
| Fe | 43400 | 43700 | 101 |
| Mn | 324 | 331 | 102 |
| Mo | 2.78 | 2.67 | 96 |
| Ni | 46.90 | 48.5 | 103 |
| Pb | 21.10 | 20 | 95 |
| Sb | 1.02 | 1 | 98 |
| Zn | 159 | 168 | 106 |

**Table S3.** Categories of pollution and risk based on EF, I_geo_, mC_d_, and PERI indexes.

| Ranges of indexes | Pollution/risk level |
| --- | --- |
| *Enrichment factor (EF)* | |
| < 2 | Depletion to minimal enrichment |
| 2-5 | Moderate enrichment |
| 5-20 | Significant enrichment |
| 20-40 | Very high enrichment |
| > 40 | Extreme enrichment |
| *Geo accumulation index (I_geo_)* | |
| < 0 | Unpolluted |
| 0-1 | Unpolluted to moderately polluted |
| 1-2 | Moderately polluted |
| 2-3 | Moderately to strongly polluted |
| 3-4 | Strongly polluted |
| 4-5 | Strongly to extremely strongly polluted |
| > 5 | Extremely polluted |
| *Modified degree of contamination index (mC_d_)* | |
| < 1.5 | Zero to very low degree of contamination |
| 1.5-2 | Low degree of contamination |
| 2-4 | Moderate degree of contamination |
| 4-8 | High degree of contamination |
| 8-16 | Very high degree of contamination |
| 16-32 | Extremely high degree of contamination |
| > 32 | Ultra high degree of contamination |
| *Potential ecological risk index (PERI)* | |
| < 150 | Low ecological risk |
| 150-300 | Moderate ecological risk |
| 300-600 | Considerable ecological risk |
| > 600 | Very high ecological risk |

| $\mathrm{ADD}_{\mathrm{ing}}= C \times{(IngR \times EF \times ED}/{BW \times AT)} \times{10}^{-6}$ | Eq. S1 |
| --- | --- |
| $\mathrm{ADD}_{\mathrm{inh}}= C \times{(InhR \times EF \times ED}/{PEF \times BW \times AT)}$ | Eq. S2 |
| $\mathrm{ADD}_{\mathrm{dermal}}= C \times{(SL \times SA \times ABS \times EF \times ED}/{BW \times AT}) \times{10}^{-6}$ | Eq. S3 |

**Table S4.** Definitions and values of parameters used to estimate the ADD of PTMs.

| Codes | Descriptions | Units | Age class | | Reference |
| --- | --- | --- | --- | --- | --- |
|  |  |  | Child | Adult |  |
| C | Concentration of elements | mg/kg | - | - | Present study |
| IngR | Ingestion rate | mg/day | 200 | 100 | (USEPA 2001) |
| InhR | Inhalation rate | m^3^/day | 7.63 | 20 |  |
| PEF | Particle emission factor | m^3^/kg | 1.36 × 10^9^ | 1.36 × 10^9^ | (USEPA 2001) |
| BW | Average body mass | kg | 15 | 70 | (USEPA 1989) |
| ED | Exposure duration | yr | 6 | 24 | (USEPA 2001) |
| EF | Exposure frequency | day/yr | 180 | 180 | * |
| SA | Exposures skin area | cm^2^ | 2800 | 5700 | (USEPA 2001) |
| AF | Skin adherence factor | mg/cm^2^.day | 0.2 | 0.07 | (USEPA 2011) |
| ABF | Dermal absorption factor | - | 0.001 | 0.001 | (USEPA 2007) |
| AT | Average time | day | ED × 365 for non-cariogenic | | (USEPA 1989) |
|  |  |  | 70 × 365 for cariogenic | |  |

* (site specific)

**Table S5**.The reference dose (RfD) and slope factor (SF) values of PTMs in this study.

|  | As | Cd | Co | Cr | Cu | Ni | Pb | Zn | Mo | Sb |
| --- | --- | --- | --- | --- | --- | --- | --- | --- | --- | --- |
| RfD_ing_ | 3.00E-04 | 1.00E-03 | 2.00E-02 | 3.00E-03 | 4.00E-02 | 2.00E-02 | 3.50E-03 | 3.00E-01 | 5.00E-03 | 4.00E-04 |
| RfD_inh_ | 3.01E-04 | 1.00E-03 | 5.71E-06 | 2.86E-05 | 4.02E-02 | 2.06E-02 | 3.52E-03 | 3.00E-01 | 4.95E-03 | - |
| RfD_dermal_ | 1.23E-04 | 1.00E-05 | 1.60E-02 | 6.00E-05 | 1.20E-02 | 5.40E-03 | 5.25E-04 | 6.00E-02 | 1.90E-03 | 8.00E-06 |
| SF_ing_ | 1.50E+00 | - | - | 5.00E-01 | - | - | 8.50E-03 | - | - | - |
| SF_inh_ | 1.51E+01 | 6.3E+00 | 9.80E+00 | 4.20E+01 | - | 8.4E-01 | 4.20E-02 | - | - | - |
| SF_dermal_ | 3.66E+00 | - | - | - | - | - | 8.5E-06 | - | - | - |

| $\mathrm{LADD}_{i}={(C \times EF}/{AT)} \times\left( {{(ER}_{\mathrm{child}}\times\mathrm{ED}_{\mathrm{child}}}/{\mathrm{BW}_{\mathrm{child}})}+{(\mathrm{ER}_{\mathrm{adult}}\times\mathrm{ED}_{\mathrm{adult}}}/{\mathrm{BW}_{\mathrm{adult}})} \right)$ | Eq. S4 |
| --- | --- |
| $\mathrm{ER}_{\mathrm{ing}}$ = IngR $\times$ 10^-6^ |  |
| $\mathrm{ER}_{\mathrm{inh}}$ = InhR/PEF |  |
| $\mathrm{ER}_{\mathrm{dermal}}$ = SA $\times$ AF $\times$ ABF $\times$ 10^-6^ |  |

**Table S6.** Statistical summary of calculated values for EF and I_geo_.

|  | As | Cd | Co | Cr | Cu | Mn | Mo | Ni | Pb | Sb | Zn | Fe |
| --- | --- | --- | --- | --- | --- | --- | --- | --- | --- | --- | --- | --- |
| **EF** | | | | | | | | | | | | |
| Average | 13.43 | 4.93 | 0.77 | 1.13 | 3.52 | 1.10 | 2.51 | 0.77 | 35.13 | 38.56 | 13.45 | - |
| Min | 4.93 | 1.25 | 0.46 | 0.62 | 0.83 | 0.57 | 0.70 | 0.34 | 8.26 | 4.13 | 2.21 | - |
| Max | 28.89 | 25.83 | 1.15 | 2.49 | 11.86 | 1.51 | 4.62 | 1.92 | 140.78 | 227.04 | 43.75 | - |
| **Igeo** | | | | | | | | | | | | |
| Average | 2.57 | 0.66 | -1.53 | -1.03 | 0.44 | -1.03 | 0.11 | -1.56 | 3.46 | 3.14 | 2.39 | -1.13 |
| Min | 1.90 | -0.26 | -2.06 | -1.77 | -0.97 | -1.37 | -0.91 | -2.35 | 1.53 | 1.47 | 0.65 | -1.61 |
| Max | 3.62 | 4.00 | -0.53 | 0.42 | 2.19 | 0.03 | 1.53 | -0.44 | 6.49 | 7.38 | 4.56 | -0.40 |

**Table S7.** Rotated component matrix for PTMs.

| PTMs | Component | | | | | |
| --- | --- | --- | --- | --- | --- | --- |
|  | 1 | | 2 | | 3 | |
| As | **0.69** | 0.31 | | 0.06 | |  |
| Cd | **0.90** | 0.14 | | 0.16 | |  |
| Co | 0.40 | **0.86** | | 0.09 | |  |
| Cr | 0.05 | **0.92** | | -0.08 | |  |
| Cu | **0.60** | -0.22 | | -0.49 | |  |
| Fe | 0.49 | **0.70** | | 0.41 | |  |
| Mn | 0.26 | -0.07 | | **0.76** | |  |
| Mo | **0.87** | 0.35 | | 0.03 | |  |
| Ni | 0.34 | **0.66** | | -0.43 | |  |
| Pb | **0.70** | **0.60** | | 0.17 | |  |
| Sb | **0.87** | 0.23 | | 0.10 | |  |
| Zn | **0.78** | 0.41 | | 0.05 | |  |

(PCA loadings >0.6 are shown in bold).

**Table S8.** The average of hazard index (HI) calculated for PTMs of Qom’s windowsill dust.

|  |  | As | Cd | Co | Cr | Cu | Mn | Ni | Pb | Zn | Mo | Sb |
| --- | --- | --- | --- | --- | --- | --- | --- | --- | --- | --- | --- | --- |
| Residential | Child | 3.27E-01 | 2.79E-03 | 3.95E-03 | 1.45E-01 | 2.26E-02 | 1.17E-01 | 1.10E-02 | 1.97E-01 | 9.54E-03 | 2.50E-03 | 3.54E-02 |
|  | Adult | 3.52E-02 | 3.26E-04 | 5.84E-04 | 1.65E-02 | 2.43E-03 | 1.69E-02 | 1.19E-03 | 2.12E-02 | 1.03E-03 | 2.68E-04 | 4.00E-03 |
| Commercial | Child | 3.20E-01 | 3.71E-03 | 3.88E-03 | 1.83E-01 | 2.64E-02 | 1.12E-01 | 1.40E-02 | 4.19E-01 | 1.41E-02 | 3.36E-03 | 7.53E-02 |
|  | Adult | 3.44E-02 | 4.35E-04 | 5.73E-04 | 2.08E-02 | 2.84E-03 | 1.62E-02 | 1.51E-03 | 4.53E-02 | 1.52E-03 | 3.62E-04 | 8.49E-03 |
| Greenspace | Child | 2.74E-01 | 2.52E-03 | 3.84E-03 | 1.63E-01 | 8.46E-03 | 1.30E-01 | 1.00E-02 | 2.17E-01 | 4.96E-03 | 1.75E-03 | 1.71E-02 |
|  | Adult | 2.94E-02 | 2.96E-04 | 5.68E-04 | 1.86E-02 | 9.10E-04 | 1.89E-02 | 1.08E-03 | 2.34E-02 | 5.35E-04 | 1.88E-04 | 1.93E-03 |
| Industrial | Child | 4.97E-01 | 1.54E-02 | 7.48E-03 | 3.00E-01 | 2.13E-02 | 1.17E-01 | 1.69E-02 | 1.73E+00 | 2.87E-02 | 5.96E-03 | 2.98E-01 |
|  | Adult | 5.34E-02 | 1.80E-03 | 1.11E-03 | 3.42E-02 | 2.29E-03 | 1.70E-02 | 1.82E-03 | 1.87E-01 | 3.10E-03 | 6.41E-04 | 3.36E-02 |

**Table S9.** The average of As, Cr and Pb carcinogenic risk (CR) in all functional zones for children and adults.

|  | As | | | | Pb | | | | Cr | | | |
| --- | --- | --- | --- | --- | --- | --- | --- | --- | --- | --- | --- | --- |
|  | Residential | Commercial | Greenspace | Industrial | Residential | Commercial | Greenspace | Industrial | Residential | Commercial | Greenspace | Industrial |
| Inhalation | 1.15E-08 | 1.12E-08 | 9.60E-09 | 1.74E-08 | 2.21E-10 | 4.72E-10 | 2.44E-10 | 1.95E-09 | 1.24E-07 | 1.57E-07 | 1.40E-07 | 2.58E-07 |
| Ingestion | 1.79E-05 | 1.75E-05 | 1.50E-05 | 2.72E-05 | 7.04E-07 | 1.50E-06 | 7.76E-07 | 6.19E-06 | 2.32E-05 | 2.94E-05 | 2.62E-05 | 4.83E-05 |
| Dermal | 1.38E-07 | 1.35E-07 | 1.15E-07 | 2.09E-07 | 2.22E-12 | 4.73E-12 | 2.45E-12 | 1.95E-11 |  |  |  |  |
| Overall | 1.81E-05 | 1.76E-05 | 1.51E-05 | 2.74E-05 | 7.04E-07 | 1.50E-06 | 7.76E-07 | 6.19E-06 |  |  |  |  |
